# Social Functioning of Childhood Cancer Survivors after Computerized Cognitive Training: A Randomized Controlled Trial

**DOI:** 10.3390/children6100105

**Published:** 2019-09-27

**Authors:** Leanne K. Mendoza, Jason M. Ashford, Victoria W. Willard, Kellie N. Clark, Karen Martin-Elbahesh, Kristina K. Hardy, Thomas E. Merchant, Sima Jeha, Fang Wang, Hui Zhang, Heather M. Conklin

**Affiliations:** 1 St. Jude Children’s Research Hospital, Memphis, TN 38105, USAjason.ashford@stjude.org (J.M.A.); victoria.willard@stjude.org (V.W.W.); kellienclark@gmail.com (K.N.C.); karenmartin1010@gmail.com (K.M.-E.); thomas.merchant@stjude.org (T.E.M.); sima.jeha@stjude.org (S.J.); fang.wang@stjude.org (F.W.); hui.zhang@stjude.org (H.Z.); 2 Children’s National Medical Center, Washington, DC 20010, USA; kkhardy@childrensnational.org

**Keywords:** attention, brain tumors, childhood cancer, computerized cognitive training, executive functioning, late effects, leukemia, social skills, social functioning, survivors

## Abstract

Childhood cancer survivors are at risk for cognitive and social deficits. Previous findings indicate computerized cognitive training can result in an improvement of cognitive skills. The current objective was to investigate whether these cognitive gains generalize to social functioning benefits. Sixty-eight survivors of childhood cancer were randomly assigned to a computerized cognitive intervention (mean age 12.21 ± 2.47 years, 4.97 ± 3.02 years off-treatment) or waitlist control group (mean age 11.82 ± 2.42 years, 5.04 ± 2.41 years off-treatment). Conners 3 Parent and Self-Report forms were completed pre-intervention, immediately post-intervention and six-months post-intervention. Piecewise linear mixed-effects models indicated no significant differences in Peer Relations between groups at baseline and no difference in change between groups from pre- to immediate post-intervention or post- to six-months post-intervention (*ps* > 0.40). Baseline Family Relations problems were significantly elevated in the control group relative to the intervention group (*p* < 0.01), with a significantly greater decline from pre- to immediate post-intervention (*p* < 0.05) and no difference in change between groups from post- to six-months post-intervention (*p* > 0.80). The study results suggest cognitive gains from computerized training do not generalize to social functioning. Training focused on skill-based social processing (e.g., affect recognition) may be more efficacious.

## 1. Introduction

Survival rates for acute lymphoblastic leukemia (ALL) and brain tumors (BTs), the most common cancers that arise in childhood, have risen substantially in the last four decades [1]. As such, the management of late effects—the onset of difficulties that arise months to years beyond the initial diagnosis and treatment—has become increasingly vital. Survivors of childhood ALL and BTs are at risk for cognitive deficits associated with both the disease itself and toxic effects of treatment [2]. The most common cognitive impairments arise in areas of visual-spatial reasoning, motor functioning, processing speed, working memory (i.e., holding in mind and manipulating information), attention, and executive functioning (i.e., higher-order thinking skills involving planning, organization, inhibition, and other goal-directed behaviors) [3,4,5]. Social-cognitive skills deficits have also been found to emerge as late effects of childhood cancer, such as poor recognition of facial expressions and social problem solving difficulties [6]. Such deficits appear to play a key role in overall social functioning problems that occur during survivorship, including poor quality of interactions, lack of close friendships, relationship problems, peer rejection, isolation, and delays in achieving social milestones [6,7,8,9]. Cognitive and social functioning late effects are also associated with reduced quality of life due to long-term functional impairments in independent living, as well as in academic, social, and vocational settings [5,7,10]. Additionally, as soon as five years post-treatment, childhood cancer survivors have been found to experience low self-esteem related specifically to their school performance and academic problems, which may further contribute to overall social functioning difficulties [11].

Demographic factors associated with greater risk for cognitive and social functioning late effects include younger age at the time of diagnosis/treatment and female sex [12,13,14]. Clinical factors play a role as well, with a BT diagnosis associated with higher risk as compared to an ALL diagnosis [3]. This is likely, in part, related to disease factors, as the size and localization of the tumor in the central nervous system (CNS) play a role in the severity of problems [15]. Additionally, CNS-directed treatments used to treat ALL and BTs, including surgical resection, chemotherapy, and radiation therapy, have all been found to increase risk for cognitive and social skills impairments, with time since treatment associated with worse outcomes [15,16,17,18]. Intrathecal chemotherapies and cranial irradiation are associated with neurotoxicity (e.g., white matter damage), which elevate the risk of poorer cognitive and social outcomes in survivorship [19,20,21,22]. While ALL is typically treated with a chemotherapy regimen, BT treatment may additionally involve surgery and cranial irradiation, posing a greater risk for BT survivors. Medical complications related to diagnosis and treatment can also put survivors at further risk for late effects, including surgical complications, the presence of increased intracranial pressure/hydrocephalus, shunt placement, seizure, and stroke [19,23].

Studies have investigated the relationship between cognitive and social skills deficits as they link to late effects of childhood cancer. Children treated for cancer who have lower overall intellectual ability have been found to exhibit poorer social information processing and social skills [24,25]. The importance of attentional control and executive functioning for age-appropriate social skills has also been highlighted [7,26]. Children with insults to the brain (e.g., as a result of diagnosis or treatment) have been found to have an impaired ability to process social stimuli (e.g., affect, facial expressions) due to attentional difficulties, and these impairments are associated with lower overall social functioning [25,27,28]. Executive functioning skills have also been found to both moderate and mediate the relationship between social-emotional interventions and social development, such that children with executive functioning deficits have more difficulty inhibiting impulses, problem solving, and attending to intervention curricula at baseline [29]. These children may also make less socio-emotional gains than other participants of an intervention as a result [29].

As reviewed by Willard [30], interventions aimed at targeting social skills directly to improve social functioning in childhood cancer survivors are typically implemented in a group format and target skills such as assertiveness, making friends, coping with rejection, conversation skills, conflict resolution, social initiation, and managing teasing/bullying. Social interventions with this population are in the early stages of development with limited success. However, interventions aimed at improving cognitive skills may generalize to social skills benefits and improved overall social functioning given the potential relationship between intellectual functioning, attention, and executive functioning with social skills. For example, prior studies have shown that methylphenidate, a stimulant medication, results not only in improved attention, but also social functioning in the classroom amongst survivors of ALL and BTs [31]. It is important to investigate this indirect approach to improving social skills, given limited support for existing direct social interventions for ALL and BT survivors.

Computerized cognitive training, which employs computer games with the aim of increasing specific cognitive skills through repetition of exercises, in conjunction with expert coaching, is a portable, safe, and time-efficient form of cognitive remediation [32]. *Cogmed*^®^ is a software program created by neuroscientists and game developers at the Karolinska Institute and involves rotating exercises designed to train visual-spatial and verbal-working memory; importantly, the difficulty level increases or decreases based on performance. It is a well-researched computerized working memory training program that has been found efficacious in improving attention, working memory, and executive functioning in populations experiencing attention difficulties [33,34]. Additionally, it has been found to be feasible and acceptable with childhood cancer survivors, with participants exhibiting high satisfaction with the training [35,36]. Accordingly, the primary objective of this study was to investigate whether previously published cognitive benefits of *Cogmed*^®^ generalize to social functioning benefits in children with ALL and BTs [32,37]. Based on the current cognitive and social skills research among childhood cancer survivors, we hypothesized that social functioning benefits would occur acutely, as well as be maintained over six-months following participation in the *Cogmed^®^* intervention. We also hypothesized that participants with a BT would experience a greater benefit as compared to those with ALL, given greater risk for social functioning deficits found in BT survivors that allow for greater improvement following intervention.

## 2. Materials and Methods

### 2.1. Participants

The current investigation is part of a larger study evaluating the efficacy of computerized cognitive training in survivors of childhood cancer. Methods have previously been described [32,37] and are summarized here. This study utilized a randomized, single-blind, waitlist-controlled, parallel-group design. Eligible participants included English-speaking survivors of childhood BTs or ALL between the ages of 8 and 16 who received cranial irradiation and/or intrathecal chemotherapy and were off-treatment for at least 1 year (without disease recurrence). Participants with IQ < 70 as documented in the medical record were ineligible. Additional exclusion criteria included premorbid history of CNS injury/disease (e.g., Down syndrome, traumatic brain injury, epilepsy), pre-existing attention-deficit/hyperactivity disorder (ADHD), severe motor/sensory deficit impeding valid testing or ability to complete intervention, use of psychotropic medications within two weeks of enrollment, and a psychological condition (e.g., active suicidal ideation or psychotic symptoms) precluding, or of higher precedence for treatment than, cognitive intervention. Recruitment transpired between October 2010 and November 2012, and written informed consent was obtained prior to study participation. The investigation was conducted at St. Jude Children’s Research Hospital, as approved by the institutional review board, and registered with ClinicalTrials.gov (NCT01217996). The study was completed in December 2013. The data that support the findings of this study are available from the corresponding author upon reasonable request.

### 2.2. Procedure

Patients were recruited consecutively in order of upcoming medical appointments. At first visit, eligibility was determined by presence of working memory problems using screening/pre-intervention cognitive assessment. To allow patients to qualify either based on absolute or relative difficulties, working memory problems were defined by Digit Span, Letter-Number Sequencing, or Spatial Span performance (Wechsler Intelligence Scale for Children—Fourth Edition (WISC-IV)) [38] greater than 1 SD below the normative mean or the individual’s IQ (Wechsler Abbreviated Scale of Intelligence (WASI)) [39]. Eligible participants were 1:1 group randomized to computerized training (*Cogmed^®^*; Pearson Education, Inc., London, UK; www.Cogmed.com) or waitlist control groups. Randomization was stratified by diagnosis (BT, ALL), age (8–11, 12–16), and gender. Block-randomization was performed by a computer system contained within the Biostatistics Department, and the person who completed randomization (H.C.) did not have advanced knowledge of group allocation determined by the computer algorithm. Only the assigned coach was notified of results of randomized group assignment. Individuals completing enrollment and assessment of cognitive outcomes were blind to group assignment. Documents that revealed randomization outcome or computerized training status were kept separate from the research chart until study completion to maintain blind conditions. A sample size of 30 was targeted for each group to afford 80% power to detect a medium size effect (0.65) between groups on cognitive measures at a significance level of 0.05.

Participants in the *Cogmed^®^* intervention group were asked to complete 25 at-home training sessions from the *Cogmed^®^* RM (for School-Age) program over 5–9 weeks. Training sessions were facilitated over the Internet, and participants utilized a laptop computer with sound speakers and a mouse to complete the exercises. The software guided participants through multiple rotating exercises each day. Each session lasted approximately 30 to 45 min and consisted of approximately eight visual-spatial and verbal working memory games. The exercises increased or decreased in difficulty and complexity based on performance with the aim of increasing the users’ working memory capacity. Progress in training was monitored over the Internet, and weekly coaching phone calls were provided for feedback and maintaining motivation. Additionally, an optional racing game was offered, which functioned as an immediate reward following each game of training. Five additional sessions were offered for participants demonstrating slower-than-desired progress, which was defined as index improvement <20 after 20 sessions.

Post-intervention/waitlist cognitive assessments occurred approximately 10 weeks after baseline assessment. All participants had a final cognitive assessment after six-months and control group members were offered the intervention off-study. Incentives for both groups included $10 gift cards following completion of 9, 17, and 25 sessions (or 2, 4, and 6 weeks for controls), as well as following pre-, post-, and six-month follow-up appointments.

### 2.3. Measures

Psychological examiners administered the same battery of cognitive measures and parent questionnaires at each time point. All selected measures contained age-specific norms from representative standardization samples and had demonstrated validity and reliability. Age-standardized abbreviated IQ was obtained from the WASI (1999) Vocabulary and Matrix Reasoning subtests. Performance-based working memory measures included the WISC-IV Spatial Span, Digit Span, and Letter-Number Sequencing tasks [38]. Parent and self-reported attention and executive functioning were measured by the Conners 3 [40].

The Conners 3 was also utilized to evaluate participants’ social functioning. The Parent Form contains 110 items rated on a scale from 0 (not true at all) to 3 (very much true). The Self-Report Form contains 99 items on the same scale. Internal consistency reliabilities range from 0.85 to 0.94 for the Parent Form and 0.84 to 0.92 for the Self-Report Form [40]. Scaled scores for both forms are age- and gender-standardized with a mean of 50 and standard deviation 10, with higher scores indicative of increased problems related to each scale. Primary scales of interest for this investigation included the parent-reported Peer Relations and self-reported Family Relations. The Peer Relations Content Scale contains 6 items (e.g., Does not get invited to play or go out with others; Has trouble keeping friends) and the Family Relations Content Scale is comprised of 8 items (e.g., My parents are too critical of me; My parents do not really care about me). Convergent validity is demonstrated with a moderate correlation between the Conners 3 Peer Relations scale and Behavior Assessment System for Children, Second Edition (BASC-2) Social Skills scale (r = −0.35 to −0.57) [40] and small to moderate correlation between the Conners 3 Family Relations scale and BASC-2 Relations with Parents scale (r = −0.15 to −0.56) [41], with negative correlations in the expected direction due to BASC-2 scales measuring adaptive behaviors where higher scores are better.

### 2.4. Statistical Analyses

Group similarity was investigated using descriptive statistics to compare demographic and clinical variables between intervention and waitlist control groups. Piecewise linear mixed-effects models were used to evaluate baseline functioning and change over time in each group. Separate slope estimates indicated group change between baseline and immediate post-intervention/waitlist cognitive assessment, as well as group change between immediate post-intervention and six-months post-intervention/waitlist cognitive assessment for outcome measures. Modeling allowed for evaluating the direction and significance of change in slope to make comparisons within groups, as well as compare the difference between groups. The models included all available data for each time point. Piecewise linear mixed-effects models were also used to evaluate potential differences in social functioning over time within diagnostic groups (ALL/BT).

## 3. Results

### 3.1. Participants

Participant enrollment and study participation have previously been described [37] and are summarized here. Study recruitment was conducted from October 2010 to November 2012, and the study was completed in December 2013. Among 128 patients screened, 80 qualified for the study based on evidence of working memory problems. Five patients were excluded, and seven declined to participate. Sixty-eight were randomized to either the control or intervention group (34 in each). Within the intervention group 30 (88%) completed at least 20 of the 25 sessions, consistent with a priori criterion for compliance [33,35]. All returned for post-intervention assessments. Within the control group, 32 returned for post-waitlist assessment and 30 for six-month post-waitlist assessment. Twenty-three initiated the intervention off-study.

Among the overall sample, participants were of generally equal gender distribution (53% male), predominantly Caucasian (78%), and largely of middle-class socioeconomic status (Table 1). Approximately two-thirds (69%) were treated for ALL, usually with chemotherapy alone (87%). Most BT participants had been given radiation therapy (73%). The average age at testing was 12 years, and participants were an average of 5 years from completion of treatment at study enrollment. Intervention and control groups were well-balanced with regard to gender, age at testing, and diagnosis, with no significant group differences in socioeconomic status, age at diagnosis, time since treatment, or intensity of treatment.

### 3.2. Prior Cognitive Findings

**Figure 1:**
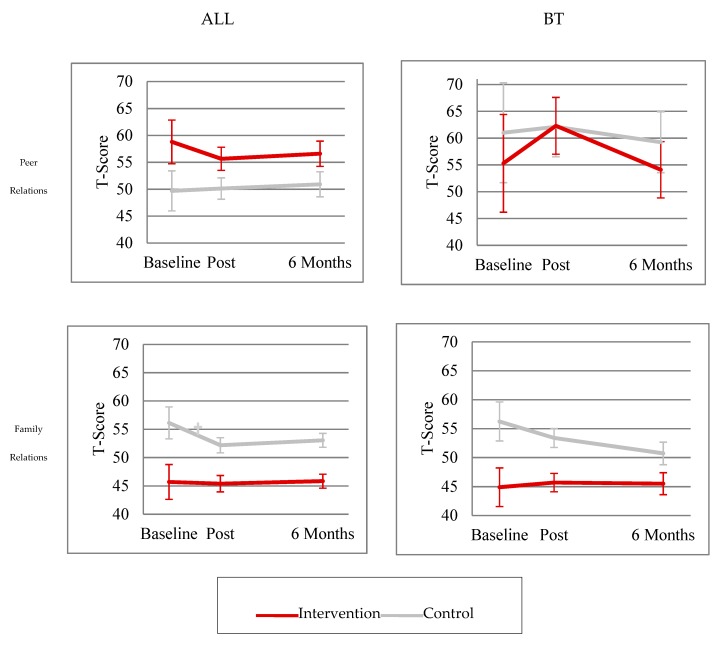
Change in Conners 3 social functioning outcomes over time in ALL vs. BT patients. ALL = acute lymphoblastic leukemia. BT = brain tumor. † Slope vs. 0.

Table 2 includes group means and standard errors for cognitive and social functioning outcomes at time points examining baseline, acute change (immediate post-intervention), and maintenance of change (six-months post-intervention). Results regarding cognitive outcomes have previously been reported and are briefly summarized here [32,37]. Piecewise linear mixed-effects models indicated no significant baseline differences between the control and intervention groups. Both groups had significantly higher parent-reported attention and executive functioning difficulties than normative expectations (Conners 3 Inattention and Executive Function Scales; *ps* < 0.05). From baseline to immediate post-intervention assessment, the intervention group demonstrated significantly greater improvement than the control group on multiple measures of attention and working memory (WISC-IV Spatial Span Forward, Digit Span Backward, Working Wemory Index, *p* < 0.05; WISC-IV Spatial Span Backward *p* < 0.001; Table 2). Parents of intervention group participants reported greater reduction in attention and executive functioning problems than parents of controls (Conners 3 Inattention and Executive Function Scales; *ps* < 0.05; Table 2). In the time between immediate post- and six-month post-assessments, intervention group performance on cognitive measures (WISC-IV Spatial Span Backward, Digit Span Backward, Working Memory Index; *ps* = 0.23–0.95; Table 2), as well as on parent-reported ratings of attention and executive functioning (Conners 3 Inattention and Executive Function Scales; *ps* = 0.62–0.69; Figure 1), remained stable.

### 3.3. Baseline Social Functioning

Baseline parent-reported Peer Relations problems were not significantly elevated in either the control or intervention group relative to normative expectations (Conners 3 Peer Relations Scale; *ps* = 0.06–0.43). There was also no significant difference in Peer Relations difficulties between the intervention and control group (*p* = 0.41). 

**Figure 2:**
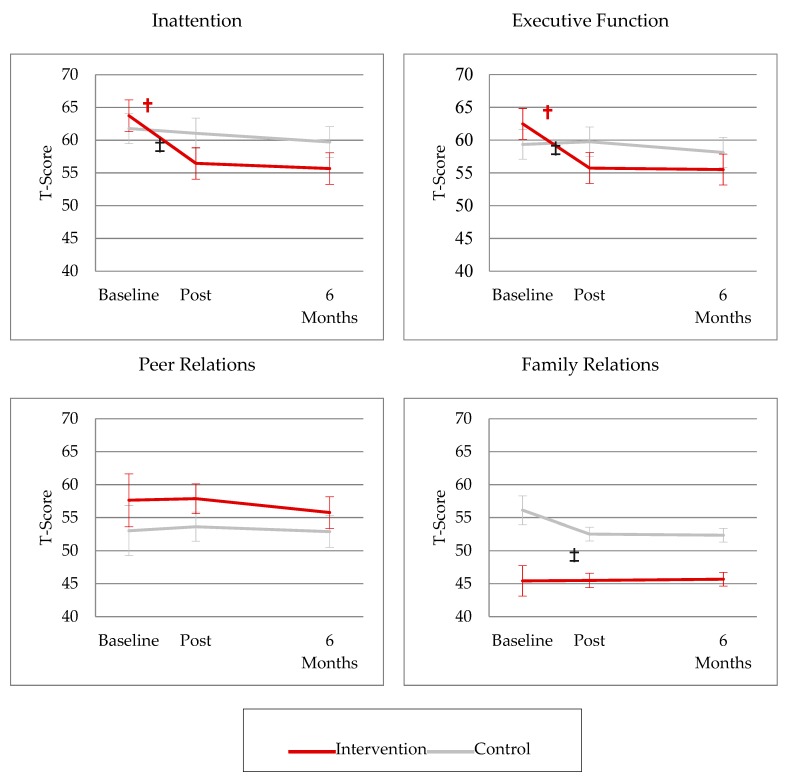
Change in Conners 3 scores over time. † Slope vs. 0. ‡ Intervention vs. Control (*p* < 0.05).

Baseline self-reported Family Relations problems were significantly elevated relative to normative expectations in the control group (Conners 3 Family Relations Scale; *p* < 0.01), but were not significantly elevated in the intervention group (*p* = 0.05). The control group also reported significantly greater Family Relations difficulties than the intervention group (*p* < 0.01; Figure 2).

### 3.4. Post-Intervention Acute Change in Social Functioning

Neither the control or intervention groups showed significant change in parent-reported Peer Relations during the period between pre- and post-immediate assessment (Conners 3 Peer Relations; *ps* = 0.78–0.92; Figure 2). There also was no significant change over this time period between groups (*p* = 0.91; Figure 2).

Control group participants demonstrated a significant decline pre- to post-waitlist with regard to self-reported Family Relations difficulties (Conners 3 Family Relations; *p* < 0.01; Figure 2) and greater relative change over this time period compared to the intervention group (*p* < 0.05). However, the intervention group showed no significant change pre- to post-intervention (*p* = 0.95; Figure 2).

### 3.5. Post-Intervention Maintenance of Change in Social Functioning

Peer Relations difficulties in neither the control or intervention groups changed significantly between immediate post- to six-months post-assessment (Conners 3 Peer Relations; *ps* = 0.38–0.76; Figure 2). There was also no difference in change over this time period between groups (*p* = 0.69; Figure 2). 

Neither the control or intervention groups showed change in Family Relations problems between immediate post- to six-months post-assessment (Conners 3 Family Relations; *ps* = 0.87–0.86; Figure 2), with no difference in change over this time period between groups (Conners 3 Family Relations; *p* = 0.81).

### 3.6. Patterns of Social Functioning by Acute Lymphoblastic Leukemia vs. Brain Tumor Diagnoses

Within the ALL group, intervention participants had significantly greater parent-reported Peer Relations problems relative to normative expectations at baseline (Conners 3 Peer Relations; *p* = 0.04), but did not differ significantly from the control group (*p* = 0.11; Figure 1). Across time points, there were no significant changes within or between control and intervention groups in Peer Relations (*ps* > 0.10; Figure 1) for those with ALL.

Among participants diagnosed with a BT, neither the intervention or the control group had significant baseline Peer Relations problems compared to normative expectations (*ps* > 0.30). There were no significant differences in Peer Relations difficulties between the control and intervention groups at baseline or with respect to change over time in those with a BT (*ps* > 0.10; Figure 1). 

Of those participants diagnosed with ALL, the control group had significantly higher self-reported Family Relations problems relative to normative expectations (Conners 3 Family Relations; *p* = 0.04) and relative to the intervention group (*p* = 0.02; Figure 1) at baseline. Among those with ALL, the control group also had a significant decline in reported Family Relations difficulties from pre- to immediate-post waitlist assessment (*p* < 0.01), but the change was not significantly different from the intervention group (*p* = 0.07; Figure 1). There were no changes within or between groups from the immediate post- to the six-months post-assessment time point when examining the ALL group.

Within the BT group, neither the intervention nor the control group had significantly elevated Family Relations problems when compared to the normative group (*ps* > 0.10). The control group had significantly higher Family Relations problems relative to the intervention group (*p* = 0.03) at baseline among those with a BT. However, there were no significant changes within or between groups over time (*ps* > 0.10; Figure 1) when examining participants diagnosed with a BT.

## 4. Discussion

*Cogmed*^®^ is a feasible and acceptable intervention for childhood cancer survivors [36], which has been found to be efficacious for targeting cognitive late effects that arise as a result of diagnosis and treatment of childhood ALL and BTs [32,35,37]. Significant improvements in children’s cognitive functioning have been demonstrated in performance-based working memory and parent-reported attention and executive functioning, with gains maintained over six-months [32,37]. 

In this study sample, neither the control nor *Cogmed*^®^ intervention group had significantly elevated parent-reported Peer Relations problems at baseline, and neither group showed acute or long-term change in Peer Relations problems through six-months post-assessment. Regarding Family Relations, waitlist controls demonstrated significantly elevated self-reported problems at baseline relative to normative expectations and relative to those in the intervention group. This group demonstrated an acute decline in Family Relations problems at immediate post-waitlist assessment, with no significant acute change noted in the intervention group. Neither group experienced a change in Family Relations over the six-month time period. There were no significant differences in change over time in either Peer or Family Relations based on an ALL or BT diagnosis.

While cognitive benefits of *Cogmed*^®^ did not generalize to social functioning benefits in children with ALL or a BT in this sample, results of this study have notable implications for future research directions. Given the extant literature, it is important to consider relationships among cognitive and social skills deficits when developing and investigating interventions that target late effects occurring in childhood cancer survivors. Determining the specific skills within the domains of attention, working memory, and executive functioning that contribute significantly to particular social-cognitive skills (e.g., social problem solving, emotion processing) will aid in development of social skills interventions, including more targeted computerized cognitive training interventions. 

While methylphenidate has been found to be a well-tolerated medication intervention that provides both long-term cognitive benefits (i.e., improved attention, executive functioning, and processing speed) and social skills benefits, this pharmacological approach has lower utility with the ALL and BT population due to issues of contraindications and side effects, poor response, and parental preference for avoiding use of a stimulant [32,33]. Thus, it is vital to continue to further investigate alternative, acceptable, efficacious intervention options that decrease late effects and improve quality of life in childhood cancer survivors with minimal health-related risks.

### 4.1. Study Limitations

The current study is limited by the measures used to evaluate social functioning (i.e., Conners 3 Peer and Family Relations). While gold standards of clinical assessments, the external validity of parent and self-report rating scales is uncertain, and it has been suggested that direct observations of an individual’s social interactions and peer nominations are better indicators of how well the child is functioning socially [41]. The scales used in this study do not include items related to social skills deficits associated with childhood cancer survivorship; rather, they are reflective of either parents’ knowledge of social interactions (Peer Relations) or the child’s perception of the family dynamic (Family Relations). As such, future research evaluating the impact of interventions on social functioning may be enhanced with the use of more reliable and valid methods of assessment, such as a combination of methods with multiple informants (e.g., rater measures by parents, teachers, and peers, as well as direct observation in the child’s natural environment). Future research should also include performance measures that can more reliably and directly assess social-cognitive skills (e.g., measures of theory of mind, facial expression recognition, affect recognition) and methods that can be better operationalized, such as peer acceptance ratings to indicate how the child is functioning with regard to level of peer rejection [30]. 

A limitation also exists in the use of a cognitive intervention that directly targets working memory skills. Investigation of a computerized training program that aims to directly target social-cognitive skills should in turn improve broader overall social functioning and potentially yield results that better support the generalization of cognitive gains to social functioning benefits. Additionally, the sample included both ALL and BT survivors, who differ in typical treatment approaches and level of risk for late effects [3]. Analyses may include investigating these diagnostic groups separately, but this also reduces the sample size and subsequent power in determining effects. Research that examines these groups separately, with ample sample sizes, may lead to a stronger understanding of intervention benefits. A larger sample would also allow for investigation of potential moderating variables of the relationship between cognitive and social outcomes (e.g., gender). This study sample was also limited in that it did not have significant rater-based social functioning problems at baseline. Future studies focused on social skills interventions should target individuals with identified difficulties in this area to increase sensitivity to change. Relatedly, another methodological limitation exists in that the control and *Cogmed*^®^ intervention groups in this study were not well-matched in terms of levels of Peer Relations and Family Relations problems at the pre-intervention time point. 

### 4.2. Clinical Implications

The current study highlights the importance of screening for and assessing specific social functioning difficulties (e.g., social skills deficits, relationship problems, social withdrawal, delays in social milestones) in addition to cognitive impairments throughout the period that late effects occur in order to make appropriate recommendations for intervention. Computerized cognitive training is an option for families searching for a flexible, convenient, and nonpharmacological approach to address cognitive late effects in childhood cancer survivors. However, survivors who are more impacted by social skills deficits would benefit from alternative interventions, as *Cogmed*^®^ alone does not appear to be sufficient in improving social functioning. For those experiencing both cognitive and social skills late effects, computerized programs directly targeting skills such as attention, working memory, and executive functioning in conjunction with a specific social functioning intervention (e.g., participation in social skills group, efforts to increase opportunities for social interaction) [30] is warranted. It is possible that gains in attention, working memory, executive functioning, and processing speed from cognitive interventions may also contribute to greater gains resulting from participation in social functioning-specific interventions (e.g., social skills training groups) [30].

## Figures and Tables

**Table 1:** Participant characteristics.

			Intervention *n* = 34	Control *n* = 34	*p*
Demographic	Gender	Female	16 (47%)	16 (47%)	1.00
		Male	18 (53%)	18 (53%)	
	Race/Ethnicity	African American	1 (3%)	5 (15%)	0.39
		Asian/Pacific Islander	1 (3%)	1 (3%)	
		Caucasian	27 (79%)	26 (76%)	
		Hispanic	2 (6%)	1 (3%)	
		Other/Multiple Races	3 (9%)	1 (3%)	
	SES (BSMSS) ^1^		39.68 ± 15.37	40.46 ± 12.20	0.82
Clinical	ALL ^2^		23 (68%)	24 (71%)	1.00
	Brain Tumor		11 (32%)	10 (29%)	0.33
		Ependymoma	1 (9%)	3 (30%)	
		Glioma	2 (18%)	0 (0%)	
		Medulloblastoma/PNET	8 (73%)	7 (70%)	
	Age at Diagnosis (years)		5.15 ± 2.92	4.62 ± 2.68	0.43
	Age at Testing (years)		12.21 ± 2.47	11.82 ± 2.42	0.51
	Time since Treatment (years)		4.97 ± 3.02	5.04 ± 2.41	0.91
	Treatment Group	Chemo ^3^ Only	20 (59%)	22 (65%)	0.95
		CSI ^4^ w/or w/o Chemo ^3^	8 (24%)	7 (21%)	
		CRT ^5^ w/or w/o Chemo ^3^	3 (9%)	3 (9%)	
		Chemo ^3^ + BMT ^6^ w/or w/o TBI ^7^	3 (9%)	2 (6%)	
	WASI IQ ^8^ (Standard Score)		106.90 ± 15.74	99.85 ± 14.01	0.06

Statistics are presented as *n* (%) or mean ± standard deviation. *p*-values indicate whether group is equally distributed across sub-categories using independent *t*-test, Chi-square or Fisher’s Exact Test, as appropriate. ^1^ Barrett Simplified Measure of Social Status (BSMSS). Derived from maternal and paternal education and occupation; scores range from 8 to 66 with higher values indicative of higher socio-economic status (SES). ^2^ ALL = acute lymphoblastic leukemia. ^3^ Chemo = chemotherapy. ^4^ CSI = craniospinal irradiation. ^5^ CRT = conformal radiation therapy. ^6^ BMT = bone marrow transplant. ^7^ TBI = total body irradiation. ^8^ WASI IQ = Wechsler Abbreviated Scale of Intelligence, Intelligence Quotient.

**Table 2:** Cognitive and social functioning scores at baseline, immediate post-intervention, and six-months post-intervention.

	Mean ± SEM ^1^					
	Intervention			Control		
**Cognitive Outcome**	**Baseline**	**Immediate Post**	**Six-Months Post**	**Baseline**	**Immediate Post**	**Six-Months Post**
WISC-IV ^2^ Digit Span Forward ^5^	9.00 ± 0.46	9.93 ± 0.53	10.23 ± 0.56	8.11 ± 0.54	8.95 ± 0.54	8.84 ± 0.55
WISC-IV ^2^ Digit Span Backward ^5^	8.97 ± 0.51	11.17 ± 0.56	10.53 ± 0.54	8.58 ± 0.52	9.21 ± 0.52	9.37 ± 0.53
WISC-IV ^2^ WMI ^3,5^	95.33 ± 2.32	104.50 ± 2.25	103.37 ± 2.39	92.50 ± 2.52	96.47 ± 2.85	95.97 ± 2.40
WISC-IV ^2^ Spatial Span Forward ^5^	9.83 ± 0.61	13.13 ± 0.64	11.63 ± 0.57	8.56 ± 0.55	9.81 ± 0.55	9.97 ± 0.56
WISC-IV ^2^ Spatial Span Backward ^5^	9.50 ± 0.61	12.63 ± 0.55	12.60 ± 0.51	10.03 ± 0.49	10.789 ± 0.49	10.81 ± 0.50
Conners 3 Parent—Inattention ^6^	63.73 ± 2.53	59.47 ± 1.39	55.67 ± 2.42	61.77 ± 2.32	61.05 ± 2.32	59.72 ± 2.35
Conners 3 Parent—EF ^4,6^	62.47 ± 2.43	55.73 ± 1.57	55.50 ± 2.36	59.33 ± 2.26	59.74 ± 2.26	58.12 ± 2.29
**Social Functioning Outcome**	**Baseline**	**Immediate Post**	**Six-Months**	**Baseline**	**Immediate Post**	**Six-Months Post**
Conners 3 Parent—Peer Relations ^6^	57.63 ± 4.00	57.87 ± 2.27	55.77 ± 2.39	53.04 ± 3.79	53.61 ± 2.18	52.90 ± 2.40
Conners 3 Self—Family Relations ^6^	45.43 ± 2.31	45.50 ± 1.10	45.68 ± 1.05	56.13 ± 2.18	52.53 ± 1.06	52.36 ± 1.05

^1^ SEM = standard error of measurement. ^2^ WISC-IV = Wechsler Intelligence Scale for Children—Fourth Edition. ^3^ WMI = Working Memory Index. ^4^ EF = Executive Function. ^5^ Scaled Score: Mean = 10, standard deviation = 3, higher score is better. ^6^ T Score: Mean = 50, standard deviation = 10, higher score is worse.
